# Temporal evolution of microstructural integrity in cerebellar peduncles in Parkinson’s disease: Stage-specific patterns and dopaminergic correlates

**DOI:** 10.1016/j.nicl.2024.103679

**Published:** 2024-09-29

**Authors:** Chentao He, Rui Yang, Siming Rong, Piao Zhang, Xi Chen, Qi Qi, Ziqi Gao, Yan Li, Hao Li, Frank-Erik de Leeuw, Anil M. Tuladhar, Marco Duering, Rick C. Helmich, Rick van der Vliet, Sirwan K.L. Darweesh, Zaiyi Liu, Lijuan Wang, Mengfei Cai, Yuhu Zhang

**Affiliations:** a Department of Neurology, Guangdong Neuroscience Institute, Guangdong Provincial People’s Hospital (Guangdong Academy of Medical Sciences), Southern Medical University, Guangzhou, Guangdong Province 510080, China; b Guangzhou Key Laboratory of Diagnosis and Treatment for Neurodegenerative Diseases, Guangdong Provincial People’s Hospital, Guangdong Academy of Medical Sciences, Guangzhou 510080, China; c Guangdong Provincial Key Laboratory of Artificial Intelligence in Medical Image Analysis and Application, Guangdong Provincial People’s Hospital, Guangdong Academy of Medical Sciences, Guangzhou 510080, China; d Radboud University Medical Center, Nijmegen, Department of Neurology, Donders Institute for Brain, Cognition and Behaviour, the Netherlands; e Medical Image Analysis Center (MIAC AG) and Department of Biomedical Engineering, University of Basel, Basel, Switzerland; f Institute for Stroke and Dementia Research (ISD), LMU University Hospital, LMU Munich, Germany; g Department of Neurology, Erasmus MC, University Medical Centre Rotterdam, Rotterdam, the Netherlands; h Department of Radiology, Guangdong Provincial People’s Hospital (Guangdong Academy of Medical Sciences), Southern Medical University, Guangzhou, Guangdong Province 510080, China

**Keywords:** Parkinson’s disease, Cerebellar peduncles, Microstructural integrity, Compensation, Dopaminergic degeneration

## Abstract

**Background:**

Previous research revealed differences in cerebellar white matter integrity by disease stages, indicating a compensatory role in Parkinson’s disease (PD). However, the temporal evolution of cerebellar white matter microstructure in patients with PD (PwPD) remains unclear.

**Objective:**

To unravel temporal evolution of cerebellar white matter and its dopaminergic correlates in PD.

**Methods:**

We recruited 124 PwPD from the PPMI study. The participants were divided into two subsets: Subset 1 (n = 41) had three MRI scans (baseline, 2 years, and 4 years), and Subset 2 (n = 106) had at least two MRI scans at baseline, 1 year, and/or 2 years. Free water-corrected diffusion metrics were used to measure the microstructural integrity in cerebellar peduncles (CP), the main white matter tracts connecting to and from the cerebellum. The ACAPULCO processing pipeline was used to assess cerebellar lobules volumes. Linear mixed-effect models were used to study longitudinal changes. We also examined the relationships between microstructural integrity in CP, striatal dopamine transporter specific binding ratio (SBR), and clinical symptoms.

**Results:**

Microstructural changes in CP showed a non-linear pattern in PwPD. Free water-corrected fractional anisotropy (FAt) increased in the first two years but declined from 2 to 4 years, while free water-corrected mean diffusivity exhibited the opposite trend. The initial increased FAt in CP correlated with cerebellar regional volume atrophy, striatal dopaminergic SBR decline, and worsening clinical symptoms, but this correlation varied across disease stages.

**Conclusions:**

Our findings suggest a non-linear evolution of microstructural integrity in CP throughout the course of PD, indicating the adaptive structural reorganization of the cerebellum simultaneously with progressive striatal dopaminergic degeneration in PD.

## Highlights

• Temporal dynamics observed in cerebellar peduncle integrity during PD progression.
• Early increase in cerebellar peduncle integrity correlates with lobule volume loss.
• Cerebellar peduncle integrity initially increases with striatal dopaminergic degeneration.
• Cerebellar reorganization might occur with progressive basal ganglia dysfunction in PD.

## 1 Introduction

In recent years, there has been increasing recognition of the significance of the cerebellum in Parkinson's disease (PD) (Li et al., 2023), primarily attributed to its critical involvement in motor and non-motor symptoms (Zhong et al., 2022, Naro et al., 2022, Maiti et al., 2020). The cerebellum has anatomical and functional connections with both the basal ganglia and the cerebral cortex, playing an crucial role in brain networks (Washburn et al., 2024, Kawabata et al., 2022, Chen et al., 2022). As PD progresses, there is an increased functional recruitment of the cerebellum and the cerebellum-cortical circuit (Hallett and Wu, 2013, Meles et al., 2021, Simioni et al., 2016). Cerebellar communication with the cortex is mediated by white matter tracts, which are instrumental for the inflow and outflow of cerebellar information (Haines and Dietrichs, 2012). Previous studies have reported increased structural integrity in cerebellar white matter in PD (Taylor et al., 2018, Li et al., 2020, Xiao et al., 2021). This has led to the hypothesis that the cerebellar white matter undergoes adaptive structural reorganization to compensate for basal ganglia dysfunction caused by pathological alterations, such as dopaminergic degeneration (Washburn et al., 2024). However, the specific structural contribution of the cerebellum to PD progression remains to be elucidated.

The inferior cerebellar peduncle (ICP), middle cerebellar peduncle (MCP), and superior cerebellar peduncle (SCP) constitute the principal white matter bundles connecting the cerebellum with the other central nervous system (Haines and Dietrichs, 2012). Although prior cross-sectional studies have implied the elevated white matter integrity in the SCP in PD compared to controls (Li et al., 2020, Xiao et al., 2021, Haghshomar et al., 2022), the temporal dynamics of cerebellar white matter integrity in PD remain debated and less understood. A recent longitudinal study has shed light on this aspect by revealing that cerebellar white matter integrity in PD was initially higher than in controls at baseline, but this trend diminished at the one-year follow-up (Taylor et al., 2018). These observations indicate that the microstructural integrity in cerebellar white matter during the progression of PD may exhibit a non-linear pattern over time with a potential compensation mechanism. However, due to the cross-sectional nature or the relatively short follow-up period in prior studies, they might have limited sensitivity to detect subtle microstructural changes during PD progression. Further investigation is required to unveil the longitudinal trajectory of microstructural alterations in cerebellar white matter with the progression of PD.

In this study, our primary aim was to investigate the temporal patterns of microstructural integrity in the cerebellar peduncles in PD by utilizing repeated measurements over 4 years. Also, we examined longitudinal volume loss of cerebellar lobules and its association with microstructural integrity of the cerebellar peduncles. Finally, we investigated the relationship between the microstructural changes in cerebellar peduncles and clinical symptoms, as well as dopaminergic degeneration during the progression of PD.

## 2 Materials and methods

### 2.1 Participants

All data analyzed in this study were obtained from the Parkinson's Progression Markers Initiative (PPMI) database (https://www.ppmi-info.org/data) as of September 2022. The PPMI is a longitudinal, multicenter observational study that combines advanced imaging, clinical, and biological data to identify biomarkers of PD progression (Marek et al., 2018, Marek et al., 2011). Ethical approval for the PPMI study was obtained from the Institutional Review Board of all participating sites, and written informed consent was obtained from all participants.

At enrollment, people with Parkinson's disease (PwPD) needed to meet specific inclusion criteria. They had to be aged 30 years or older, untreated with medications commonly prescribed for PD (levodopa, dopamine agonists, MAO-B inhibitors, or amantadine), within two years of initial diagnosis, with a Hoehn and Yahr stage below 3. Additionally, they were required to exhibit either a combination of at least two of the following symptoms: resting tremor, bradykinesia, or rigidity (with an essential presence of either resting tremor or bradykinesia), or demonstrate a single asymmetric resting tremor or asymmetric bradykinesia.

**Fig. 1:**
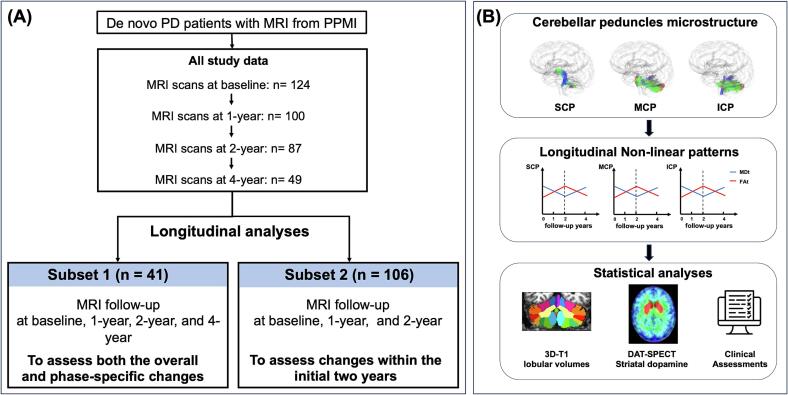
Flowchart of study design. (A) This cohort comprised 124 PwPD with both T1w and DWI scans at baseline (year 0), with subsequent scans available for 100, 87, and 49 participants at 1-year, 2-year, and 4-year follow-up, respectively. For longitudinal analyses, due to the decreasing number of participants with MRI data (both T1w and DWI) at each follow-up, the cohort was divided into Subset 1 (n = 41) and Subset 2 (n = 106). Subset 1 included participants with three MRI time points (baseline, 2 years, and 4 years), while Subset 2 included participants with at least two MRI time points among baseline, 1 year, and 2 years. Subset 1 was used to examine overall and stage-specific changes from baseline to 2 years and from 2 years to 4 years. Subset 2 was used to investigate changes within the initial 2 years to validate the robustness of the results observed in Subset 1. (B) The study assessed the microstructural integrity of the cerebellar peduncles, investigating their longitudinal changes and phase-specific variations over time, and examined how these alterations relate to cerebellar atrophy, striatal dopaminergic changes, and clinical symptoms. Superior Cerebellar Peduncle (SCP), Middle Cerebellar Peduncle (MCP), and Inferior Cerebellar Peduncle (ICP).

In this study, we specifically used T1 and diffusion-weighted imaging (DWI) data from PwPD. Only participants with both clinical assessments and baseline DWI scans were included. This cohort consisted of 124 PwPD with DWI scans at baseline (year 0), while 100, 87, 49 had DWI scans at 1-year, 2-year, 4-year follow-up, respectively. A flowchart of participants included and study design is shown in Fig. 1. Additionally, we included baseline DWI and T1 data from 45 controls; however, fewer than 10 controls had both T1 and DWI follow-up data available at years 1, 2, and 4.

Because of the decreasing number of participants with MRI data per follow-up visit, we conducted our analyses using two subsets: (i) **Subset 1** (n = 41) was used to assess both the overall and phase-specific changes in cerebellar microstructure over the course of 4 years, with MRI follow-up data available for each participant at three time points (baseline, 2-year, and 4-year), with or without 1-year data. (ii) **Subset 2 (**n = 106) consisting of a larger sample size was used to assess the robustness of the results, specifically focusing on changes occurring within the initial two years. This subset included individuals who had data at least two repeated MRI assessments among baseline, 1-year, and 2-year time points. These two subsets are derived from the same cohort, with the primary difference being the timing of MRI follow-ups. While the subsets are not entirely independent, they play the complementary roles in validating our findings. Details on the sample size and follow-up timelines for both subsets are provided in Supplementary Fig. 1. Site information and participant distribution across study subsets are provided in Supplementary Table 1.

### 2.2 Clinical assessments

All participants in the PPMI study underwent standardized assessments over a 4-year follow-up (Marek et al., 2018). In the present study, to explore clinical characteristics related to the imaging pattern changes, we utilized clinical data collected at baseline, the 2-year, and the 4-year time points. The evaluation of primary PD-related symptoms, covering Part I to Part III, was conducted using the Movement Disorder Society–sponsored revision of the Unified Parkinson’s Disease Rating Scale (MDS-UPDRS) (Goetz et al., 2008). Additionally, our study incorporated non-motor scales, such as the 15-item Geriatric Depression Scale (GDS-15), State–Trait Anxiety Inventory (STAI), Montreal Cognitive Assessment (MoCA), the Scales for Outcomes in Parkinson’s disease − Autonomic Dysfunction (SCOPA-AUT), and the REM Sleep Behavior Questionnaire (RBD). Levodopa equivalent daily dose (LEDD) for baseline and every visits were calculated (Tomlinson et al., 2010).

### 2.3 MRI data acquisition

Considering that MRI data in PPMI were obtained from various MRI manufacturers at multiple sites, to reduce the site effect and enhance the robustness of the MRI-derived metrics, we exclusively included subjects who were scanned using the same manufacturer of MRI scanner (all by Siemens 3T Tim Trio) and followed an identical MRI acquisition protocol as outlined below. 3D-T1-weighted MRI data from PPMI were acquired using 3 Tesla scanners using magnetization prepared rapid gradient echo (MPRAGE) sequences with following parameters: TE = 2.98 ms, TR = 2300 ms, TI = 900 ms, voxel size = 1 × 1 × 1 mm^3^, flip angle = 9°, matrix size = 240 mm × 256 mm, slice thickness = 1 mm. The cardiac gated spin-echo echo-planar imaging (SE-EPI) diffusion MR sequences were acquired with the following parameters: TR = 900 ms, TE = 88 ms, flip angle = 90°, in-plane resolution 2 × 2 mm^2^, slices = 72, slice thickness = 2 mm. The acquisition included 64 gradient directions with a b-value of 1000 s/mm^2^, and one reference non-gradient volume (b = 0 s/mm^2^).

### 2.4 Cerebellar volumetric analysis

Cerebellar lobules were automatically segmented using the ACAPULCO (Automatic Cerebellum Anatomical Parcellation using U-Net with Locally Constrained Optimization) algorithm (version 0.2.1; https://gitlab.com/shuohan/acapulco) (Han et al., 2020). This segmentation method employs a U-Net architecture with locally constrained optimization, trained on expert-hand segmented parcellations. The quality control process involves several steps quality checked in accordance with the standardized protocol (Kerestes et al., 2022). Since we did not consider lateralization here, right and left lobular volumes have been combined and finally 12 cerebellar regions have been studied, including lobules I–III, IV, V, VI, Crus I, Crus II, VIIb, VIIIa, VIIIb, IX, X, and one cerebellar vermis region. In order to compare volumetric measurements among individuals, the volumes of all cerebellar lobules were adjusted for variations in head size by expressing them as a percentage of the intracranial volume (ICV). Here, ICV was derived from Sequence Adaptive Multimodal SEGmentation (SAMSEG), a well-established tool to robustly segment brain structures from head MRI scans without preprocessing (Puonti et al., 2016). The workflow of MRI processing used in the present study was summarized in Supplementary Fig. 2.

### 2.5 Diffusion MRI preprocessing

DWI preprocessing steps included visual quality control, principal component analysis-based denoising, Gibbs artefact removal and dynamic correction for susceptibility-induced distortions, eddy current-induced distortions, as well as head motion using tools from MRtrix3 (https://www.mrtrix.org, dwidenoise, mrdegibbs) and eddy (Dewenter et al., 2023). In the absence of unweighted diffusion images with reversed phase-encoding in the DWI scans, we employed Synb0-DISCO to synthesize an unweighted diffusion image free from susceptibility-induced distortions. This synthesized image, derived from T1-weighted images, was used to perform the top-up step, reducing spatial distortions caused by susceptibility-induced off-resonance fields (Schilling et al., 2019).

### 2.6 Microstructural measurements of cerebellar peduncles

Microstructural integrity of cerebellar peduncles is typically quantified using diffusion MRI with the traditional single-tensor model (Haghshomar et al., 2022). However, the anatomical connection between the cerebellar peduncles and the pons, with the fourth ventricle in between, raises concerns about potential influences from cerebrospinal fluid (CSF) partial volume effects. To mitigate this concern, we employed the bi-tensor model, originally developed to correct for CSF contamination (Pasternak et al., 2009). This bi-tensor model approach could enhance the sensitivity of diffusion metrics in the cerebellar peduncles, enabling the identification of nuanced microstructural changes in the cerebellar peduncles in PD.

To quantify the microstructural changes in the cerebellar peduncles, we employed the following metrics within the bi-tensor free water (FW) model: free water fraction (FWf), free water-corrected mean diffusivity (MDt), and fractional anisotropy (FAt). In brief, the signal in each voxel was fitted to a two-compartment model, consisting of a free water (FW) compartment represented by an isotropic tensor with a fixed diffusion constant of water at 37 °C and a tissue compartment represented by a free water-corrected tensor (Duering et al., 2018). The parameters estimated were the fractional volume of the FW compartment (i.e., FWf) and the tensor of the tissue compartment, i.e., free water-corrected mean diffusivity (MDt), and fractional anisotropy (FAt). The fractional volume of the free water compartment (i.e., FWf) reflects the relative contribution of free water in each voxel, ranging from 0 to 1 (Zhou et al., 2023). Lower FAt and higher MDt values may suggest potential axonal structural damage (Basser and Pierpaoli, 1996), while higher FA or lower MD values may indicate increased axonal density in specific pathways, possibly due to axonal sprouting and reorganization (Rau et al., 2019). The free water model was estimated using a processing kit provided by the MarkVCID project (https://markvcid.partners.org/markvcid1-protocols-resources).

The superior cerebellar peduncle (SCP), middle cerebellar peduncle (MCP), and inferior cerebellar peduncle (ICP) were identified as the tracts of interest (TOI) within the cerebellum (Fig. 1). The MCP and ICP are the primary afferent pathways, with the MCP receiving input from the contralateral cerebral cortex via the pontine nuclei, and the ICP conveying signals from the olivary nucleus and spinal cord. The SCP serves as the main efferent pathway, connecting the deep cerebellar nuclei to the thalamus.

Cerebellar peduncles were segmented using tractography-based Segmentation (TractSeg) (Wasserthal et al., 2018). This novel convolutional neural network-based approach was developed to directly segment tracts in the field of fiber orientation distribution function (fODF) peaks without using tractography, image registration or parcellation and can accurately reconstruct fiber tracts in native space. Given its unprecedented accuracy over the mostly commonly employed and conventional approach of tract reconstruction, masks of cerebellar peduncles in native space across different time points were obtained separately, followed by the extraction of tract-specific diffusion metrics, including FWf, FAt and MDt.

The FWf metric denotes the relative contribution of free water in each voxel. The tensor of the tissue compartment reflects the tissue microstructure after removing the signal contributed by FW. From this tensor, the tissue compartment measures of MDt and FAt were calculated using fslmaths in FSL. For each tract in every participant, the mean values of diffusion metrics were extracted across all voxels. For each time point, these diffusion metrics were quantified in its native space. A total of 13 scans out of 373 scans (3.48 %) were excluded due to artifacts, image quality problems, and issues with cerebellar tract segmentations.

### 2.7 DAT-SPECT analysis

Repeated dopaminergic imaging using 123I-ioflupane to target the dopamine transporter (DAT-SPECT) was conducted at baseline, 1-year, 2-year, and 4-year follow-up. The processing of DAT-SPECT data followed the PPMI protocol, with outcome measures comprising striatal specific binding ratios (SBR) for both the caudate and putamen (Marek et al., 2018). The SBR values for Parkinson's disease were obtained from the PPMI website. Striatal SBR was a summative measure of putamen and caudate SBR (Costello et al., 2023).

### 2.8 Statistical analysis

Two-tailed p values of <0.05 were considered statistically significant. Unless otherwise specified, all statistical analyses were carried out in R, version 4.1.1.

### 2.9 Temporal evolution of microstructural integrity in cerebellar peduncles

To examine the longitudinal changes of microstructural integrity in cerebellar peduncles over 4 years, we used linear mixed-effects models for each diffusion metric in **subset 1**.We fitted the mixed-effect models using data from the 0–1-2 years, 2–4 years, and 0–1-2–4 years, respectively, since we hypothesized that changes in cerebellar white matter integrity measurements during the development of PD are non-linear as postulated by previous research (Li et al., 2020). The diffusion metrics served as dependent variables. The fixed effects included follow-up time (variable of interest), sex, age and sites at baseline. Random effects were included as follows: **1)** for models with 0–1–2 years and 0–1–2–4 years data (involving more than two time points), follow-up time was taken as the random effect for each participant; **2)** for the models with 2–4 years data (with only two time points), since there were not enough time points to estimate random slopes for each participant, subjects were regarded as a random factor with a random intercept (Bates et al., 2018).

Additionally, to assess the robustness of the results on changes occurring within the initial two years, we replicated the analysis of longitudinal changes in diffusion metrics over the 0–1–2 years follow-up period in **subset 2**.

In addition to these main analyses, we conducted various additional analyses. For instance, we included results from initial two-year models that also adjusted for the levodopa equivalent daily dose (LEDD) at year 2. As a sensitivity analysis, considering the variable symptom duration of PwPD at baseline (1.5 [0.9, 2.9] years), we repeated linear mixed-effects models for patients who had their baseline scan within 1.5 years of disease onset (n = 60) to confirm the FA increase over the following two years.

### 2.10 Longitudinal changes in cerebellar volume

To evaluate the internal consistency of changes in cerebellar structure and microstructure, we investigated the volume changes of cerebellar lobules over 4 years using linear mixed-effects models. The volumes of cerebellar lobules at each time point were used as the dependent variable. The fixed effects included follow-up time, sex, age, and sites at baseline. Random effects were included as follows: 1) for models with 0–1–2 years data (involving more than two time points), follow-up time was taken as the random effect for each participant; 2) for the models with 2–4 years data (with only two time points), subjects were regarded as the random intercept. In this analysis, the fixed effect of follow-up time represents the average change in cerebellar volume per follow-up interval.

To calculate the individual cerebellar atrophy rates for each participant in the initial 2 years, we extracted the random slopes of follow-up time in the models with 0–1–2 years. To calculate the cerebellar atrophy rates for each participant in 2–4 years, we subtracted the 2-year volume from the 4-year volume first, which is further divided by the duration between 2 and 4 years for individuals.

### 2.11 Relationship between cerebellar lobular volumes and cerebellar diffusion metrics

In the next step, we explored whether potentially increased white matter integrity occurred simultaneously with cerebellar atrophy in the early stage. To explore the relationship between diffusion metrics in cerebellar peduncles and the loss of cerebellar volume, we performed separate Spearman's correlations. We specifically analyzed: 1) cross-sectionally, the correlation between lobular volumes and cerebellar diffusion metrics at baseline across the entire cohort (n = 124); 2) longitudinally, the relationship between changes in cerebellar diffusion metrics and the atrophy rate of each cerebellar lobules during 0–2 years and 2–4 years in **subset 1** of the cohort (n = 41).

### 2.12 Relationships between cerebellar diffusion metrics and clinical symptoms and dopaminergic neurodegeneration

Finally, to investigate the correlations between cerebellar diffusion metrics and clinical symptoms as well as dopamine uptake rates at different stages of PD, we conducted separate Spearman's correlations at each time point.

To further explore potential nonlinear relationships between striatal DAT SBR on cerebellar diffusion metrics, we conducted the 'two-lines' test across all available contemporaneously acquired data points and years of follow-up (Simonsohn, 2018). In essence, this method uses “Robin Hood” algorithms to identify a breakpoint in the distribution and assesses whether the regression lines on each side of this breakpoint show significant slopes with opposite directions, suggesting a U-shaped pattern.

Conducting numerous statistical tests typically increases the risk of a type I error. Nevertheless, considering the exploratory nature of our study and our focus on specific regions and tracts rather than a universal hypothesis, we did not choose to reduce that probability while increasing the probability of a type II error through a smaller significance cut-off or multiple comparisons adjustment (Rothman, 1990).

## 3 Results

### 3.1 Demographic and clinical characteristics

The mean age (SD) of PwPD at baseline, 2-year follow-up and 4-year follow-up was 61.0 (9.5), 61.5 (9.0), and 58.4 (10.1) years, respectively. At baseline, 44 (35.5 %) were women. The median (IQR) symptom duration at baseline, 2-year and 4-year follow-up was 1.5 [0.9, 2.9], 3.4 [2.9, 4.8], and 5.2 [4.9, 6.2] years, respectively. Detailed demographic and clinical characteristics are shown in Table 1.

**Table 1:** Demographic and clinical characteristics of all patients with Parkinson’s disease.

	Baseline	1-year	2-year	4-year	P
n	124	100	87	49	
Female, n (%)	44 (35.5)	33 (33.0)	32 (36.8)	15 (30.6)	0.878
Age, years, mean (SD)	61.0 (9.5)	60.8 (9.5)	61.5 (9.0)	58.4 (10.1)	0.307
Education, years, median [IQR]	16.0 [14.0, 18.0]	16.0 [13.0, 18.0]	16.0 [13.0, 18.0]	16.0 [14.0, 18.0]	0.703
Age at onset, median [IQR]	59.7 [51.3, 66.2]	59.7 [51.2, 66.0]	60.0 [53.0, 66.2]	56.4 [48.8, 64.1]	0.477
Symptom duration, years, median [IQR]	1.5 [0.9, 2.9]	2.3 [1.9, 3.8]	3.4 [2.9, 4.8]	5.2 [4.9, 6.2]	<0.001
LEDD, mg, median [IQR]	0.0 [0.0, 0.0]	225.0 [15.2, 400.0]	402.5 [201.2, 604.8]	700.0 [500.0, 1032.0]	<0.001
MDS UPDRS I, median [IQR]	4.5 [2.0, 6.0]	5.0 [2.8, 8.2]	6.0 [4.0, 9.0]	7.0 [4.0, 12.0]	<0.001
MDS UPDRS II, median [IQR]	5.0 [2.0, 8.0]	7.0 [4.0, 9.0]	7.0 [4.0, 10.5]	8.0 [3.0, 11.0]	<0.001
MDS UPDRS III, median [IQR]	21.0 [14.0, 28.0]	26.0 [17.0, 30.0]	28.0 [20.2, 37.0]	26.5 [17.8, 34.2]	<0.001
Hoehn Yahr stage, median [IQR]	2.0 [1.0, 2.0]	2.0 [2.0, 2.0]	2.0 [2.0, 2.0]	2.0 [2.0, 2.0]	<0.001
MoCA, median [IQR]	28.0 [27.0, 29.0]	28.0 [25.5, 29.0]	27.0 [25.0, 29.0]	28.0 [26.0, 30.0]	0.035
GDS, median [IQR]	5.0 [5.0, 6.0]	5.0 [4.0, 6.0]	5.0 [5.0, 6.0]	5.0 [4.0, 6.0]	0.314
STAI, median [IQR]	64.0 [54.0, 75.2]	65.0 [51.5, 78.0]	66.0 [54.0, 79.5]	69.0 [54.0, 78.0]	0.906
RBD, median [IQR]	3.5 [2.0, 5.2]	3.0 [2.0, 5.0]	4.0 [2.0, 5.5]	4.0 [2.0, 7.0]	0.577
SCOPA AUT, median [IQR]	8.0 [5.0, 12.0]	10.0 [6.0, 14.0]	10.0 [6.0, 15.0]	11.0 [7.0, 15.0]	0.01
DATSCAN striatum (yes), n (%)	120 (96.8)	97 (97.0)	85 (97.7)	46 (93.9)	0.68

Data represent number of participants (%), mean (SD) or median [IQR]. LEDD = Levodopa equivalent daily dose; MDS-UPDRS = Movement Disorder Society − sponsored revision of the Unified Parkinson’s Disease Rating Scale; MoCA = Montreal Cognitive Assessment; GDS = Geriatric Depression Scale; STAI = State − Trait Anxiety Inventory; RBD = REM Sleep Behavior Questionnaire; SCOPA-AUT = Scales for Outcomes in Parkinson’s disease − Autonomic Dysfunction; DATSCAN = the dopamine transporter scan.

### 3.2 Longitudinal changes of microstructural integrity in cerebellar peduncles over time: an early increase followed by a decrease

**Fig. 2:**
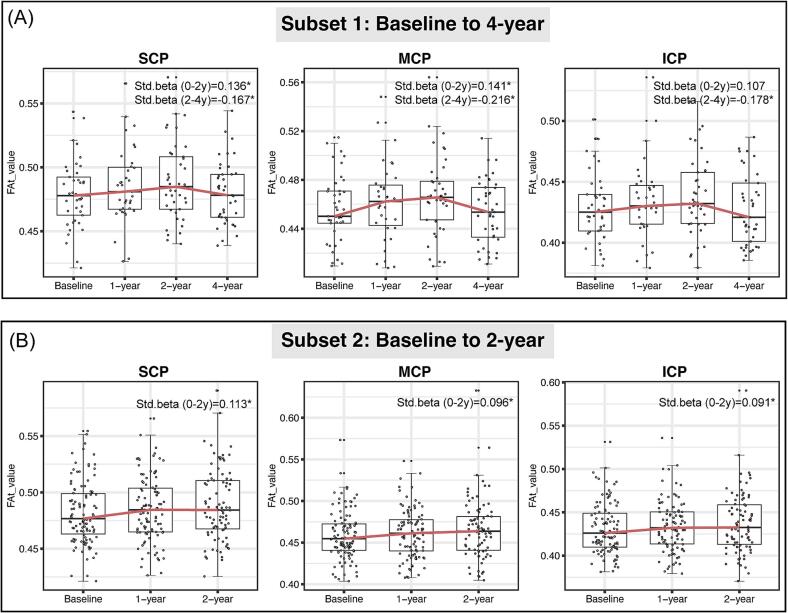
Progression patterns of microstructural integrity in cerebellar peduncles over time in PD. (A) Longitudinal changes in cerebellar peduncles microstructural integrity in subset 1 (n = 41). Standardized beta (std.beta) was extracted from liner mixed models for each period (0–2 and 2–4 years), while the boxplots were intended to visualize the changing trend for each time point. (B) Longitudinal changes in cerebellar peduncles microstructural integrity in subset 2 (n = 106). ICP = Inferior Cerebellar Peduncle; MCP = Middle Cerebellar Peduncle; SCP = Superior Cerebellar Peduncle; MDt = free water-corrected mean diffusivity; FAt = free water-corrected fractional anisotropy; Std.beta = Standardized beta coefficients. * indicates significant change.

During the 0 to 2-year follow-up period in Subset 1, FAt values increased in the MCP (β = 0.141, P = 0.011) and SCP (β = 0.136, P = 0.011) (Fig. 2A), while MDt values decreased in the MCP (β = −0.122, P = 0.024) and SCP (β = −0.126, P = 0.018) (Supplementary Table 2). Similar trends were observed in the ICP, although without statistical significance (FAt in ICP: β = 0.107, P = 0.064; MDt in ICP: β = −0.101, P = 0.054). However, there was no significant changes in FWf values. Analyses with Subset 2 also yielded similar results (Fig. 2B, Supplementary Table 2). When adjusted for LEDD, both Subset 1 and Subset 2 yielded similar results (Supplementary Table 3). Sensitivity analyses in patients with symptom duration within 1.5 years also showed similar results, confirming the FAt increase during the initial two years of follow-up (Supplementary Fig.3). With bonferroni multiple comparison corrections, the results remained similar (Supplementary Table 2).

During the 2 to 4-year follow-up period in Subset 1, FAt values decreased in the ICP (β = −0.178, P = 0.004), MCP (β = −0.216, P = 0.001), and SCP (β = −0.167, P = 0.016) (Fig. 2A), while MDt values increased in ICP (β = 0.208, P = 0.004), MCP (β = 0.233, P = 0.001), and SCP (β = 0.182, P = 0.017) (Supplementary Table 2). Notably, there were no significant changes in free water fraction during this period. All results are shown in Supplementary Table 2. Therefore, we focused on MDt and FAt in the following analyses.

When examining longitudinal changes from baseline to the 4-year follow-up, there were no significant alterations in any of the diffusion metrics in the three cerebellar peduncles (P > 0.05) (Supplementary Table 4).

### 3.3 Longitudinal cerebellar volume changes over time and their relationships with microstructural metrics in cerebellar peduncles

**Fig. 3:**
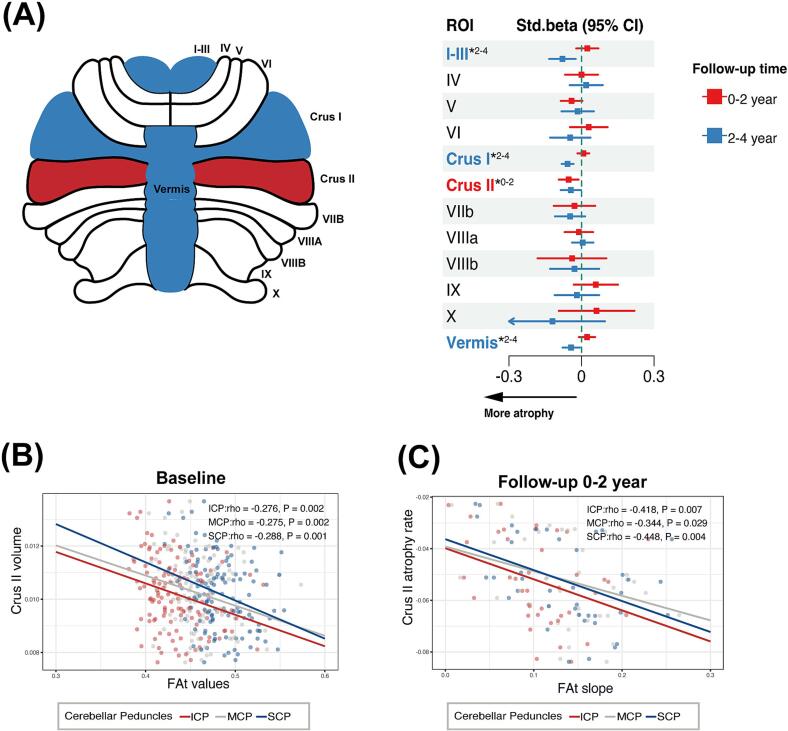
Longitudinal changes of cerebellar volumes and their association with microstructural metrics in cerebellar peduncles. (A) Longitudinal changes in cerebellar volume over 4 follow-up years in the subset 1 (n = 41). The y-axis indicates the regions of interest (ROI). The x-axis displays the rate of annual percentage volume change (standardized beta coefficients) with confidence intervals (CI), adjusted for age, gender, and site. * indicates significant atrophy. (B) At baseline (n = 124), higher microstructural integrity (higher FAt values) of cerebellar peduncles correlated with lower volumes of cerebellar lobule Crus II. (C) In subset 1 (n = 41), a faster increase in white matter integrity (higher FAt slope) in cerebellar peduncles is linked to more cerebellar lobule Crus II volume atrophy during the 0–2 years period. ICP = Inferior Cerebellar Peduncle; MCP = Middle Cerebellar Peduncle; SCP = Superior Cerebellar Peduncle; MDt = free water-corrected mean diffusivity; FAt = free water-corrected fractional anisotropy; Std.beta = Standardized beta coefficients.

To assess the internal consistency of changes in cerebellar structure and microstructural alterations, we first investigated the longitudinal changing patterns in cerebellar volumes over 4 years. We found: **1)** during the 0 to 2-year follow-up period, only lobule Crus II showed significant volume loss (β = −0.053, P = 0.014) (Fig. 3A). **2)** in the 2 to 4-year follow-up period, lobule I-III (β = −0.079, P = 0.006), lobule Crus I (β = −0.058, P < 0.001), vermis (β = −0.0425, P = 0.021) and lobule Crus II (β = −0.044, P = 0.041) showed significant volume loss (Fig. 3A).

In the next step, we explored whether potentially increased white matter integrity occurred simultaneously with cerebellar atrophy in the early stage. We observed that higher microstructural integrity was associated with volume loss in cerebellar lobules in the early stage. Specifically, **1)** cross-sectionally (n = 124, at baseline across the entire cohort), higher microstructural integrity of cerebellar peduncles correlated with lower volumes of cerebellar lobules at baseline, including I-III, Crus II, VIIb, and vermis (Fig. 3B, Supplementary Table 5). However, these correlations were not significant in the control group (n = 45) (Supplementary Fig. 4); **2)** longitudinally, in the initial 2 years in **subset 1**, progression rate of microstructural integrity in cerebellar peduncles correlated with a higher atrophy rate of cerebellar Crus II (Fig. 3C, Supplementary Table 6). Conversely, we found that lower microstructural integrity of cerebellar peduncles was correlated with lower volumes of cerebellar lobules, including VIIIa and X lobules. However, in the analyses for 2–4 year in **subset 1**, we did not observe significant correlations with the regions exhibiting notable atrophy (Supplementary Table 7).

### 3.4 Clinical associations with microstructural integrity in cerebellar peduncles at different stages

**Fig. 4:**
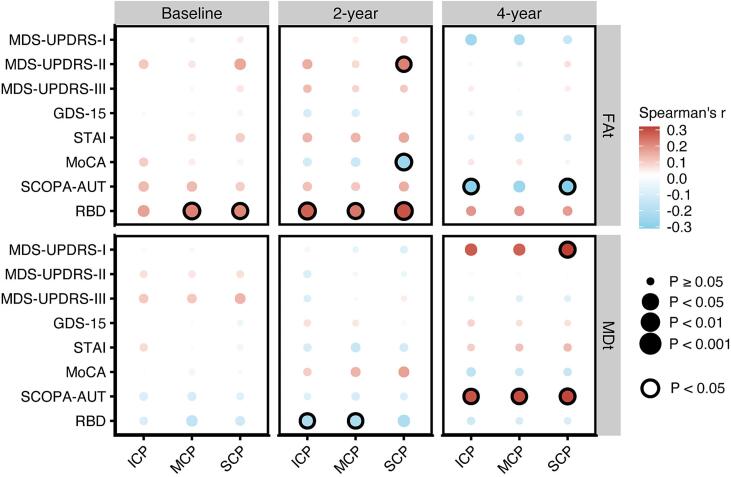
Clinical correlations with microstructural integrity in cerebellar peduncles at different follow-up time points (baseline, 2-year, and 4-year) in PD. The dot color represents the magnitude of clinical correlation, positive (red) or negative (blue), while the dot diameter denotes the P value of these correlations. Correlation estimates with P < 0.05 are annotated with solid black circles. MDS UPDRS = Movement Disorder Society − sponsored revision of the Unified Parkinson’s Disease Rating Scale; MoCA = Montreal Cognitive Assessment; GDS = Geriatric Depression Scale; STAI = State − Trait Anxiety Inventory; RBD = REM Sleep Behavior Questionnaire; SCOPA-AUT = Scales for Outcomes in Parkinson’s disease − Autonomic Dysfunction; DATSCAN = the dopamine transporter scan; ICP = Inferior Cerebellar Peduncle; MCP = Middle Cerebellar Peduncle; SCP = Superior Cerebellar Peduncle; MDt = free water-corrected mean diffusivity; FAt = free water-corrected fractional anisotropy.

Next, we tested the association between cerebellar diffusion metrics and clinical symptoms at different stages of PD. We observed a positive relationship between RBD scores and FAt in the SCP and MCP, and ICP at both baseline and the 2-year follow-up (rho range 0.21–0.29, P < 0.05), with a slightly weaker association for FAt in the ICP at baseline (rho = 0.17, P = 0.071) (Supplementary Fig. 5, Supplementary Table 8). However, these associations were no longer significant at the 4-year follow-up (Supplementary Table 8). Additionally, at the 4-year follow-up, we observed a negative correlation between SCOPA-AUT scores and the FAt values in the SCP, ICP, and MCP (rho range −0.3 to −0.31, P < 0.05, except for FAt in MCP with SCOPA-AUT score), respectively (Supplementary Fig. 5, Supplementary Table 8). Visualization of all correlation results between cerebellar diffusion metrics and clinical symptoms at baseline, 2-year, and 4-year was shown in Fig. 4.

### 3.5 Increased microstructural integrity in cerebellar peduncles correlated with striatal dopaminergic degeneration

**Fig. 5:**
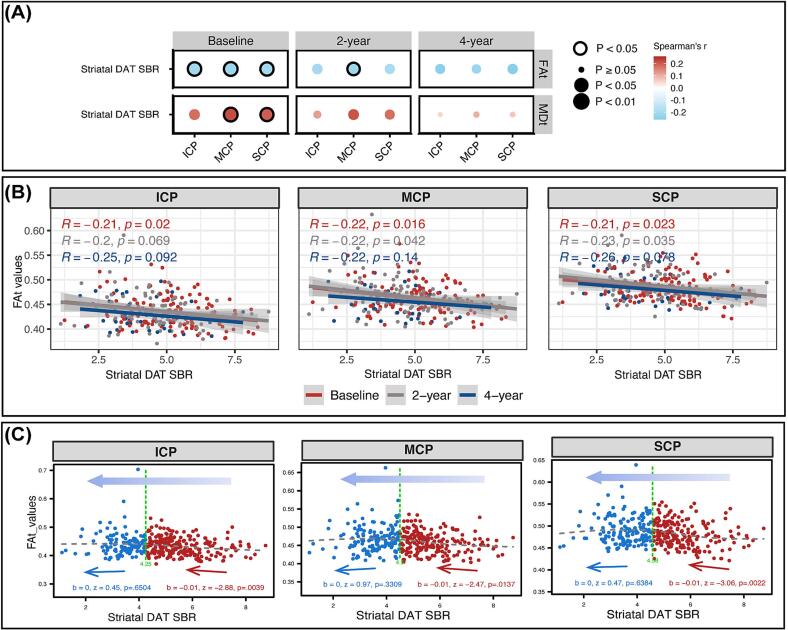
Relationship between microstructural integrity in cerebellar peduncles and striatal dopaminergic degeneration in PD. (A) Striatal dopamine transporter specific binding ratio (DAT SBR) correlations with cerebellar peduncles microstructural integrity at different follow-up time points (baseline, 2-year, and 4-year). The dot color represents the magnitude of clinical correlation, positive (red) or negative (blue), while the dot diameter denotes the P value of these correlations. Correlation estimates with P < 0.05 are annotated with solid circles. (B) Scatter plots displaying the correlations between striatal DAT SBR and FAt values in SCP, MCP, and ICP at baseline, 2-year, and 4-year. (C) Two-lines test assessing potential nonlinear relationships between striatal DAT SBR and cerebellar diffusion metrics, across all available acquired data points and years of follow-up. ICP = Inferior Cerebellar Peduncle; MCP = Middle Cerebellar Peduncle; SCP = Superior Cerebellar Peduncle; MDt = free water-corrected mean diffusivity; FAt = free water-corrected fractional anisotropy.

Finally, we investigated the relationships between cerebellar diffusion metrics and dopamine uptake rates at different stages of PD. At baseline, we observed significantly positive correlations between striatal DAT SBR and MDt values in SCP, MCP, and ICP (rho range 0.18–0.2, P < 0.05), as well as significant negative correlations with FAt values in SCP and MCP for each (rho range −0.21 to −0.22, P < 0.05) (Fig. 5A-B, Supplementary Table 9). However, these correlations weakened at the 2-year follow-up and became non-significant at the 4-year follow-up (Fig. 5A, Supplementary Table 9).

Next, we explored whether there is a threshold for striatal dopamine degeneration (DAT SBR), below which the correlation with increased white matter integrity in cerebellar peduncles becomes non-significant. Using the two-lines test, we conducted an exploratory analysis that included all available contemporaneously acquired data points over all years, further delving into the potential non-linear relationship. The estimated threshold for striatal DAT SBR values fell within the range of 4.25 to 4.58. Above this threshold, a negative relationship with FAt in all cerebellar peduncles was found (β = −0.01, P < 0.05), while below this threshold, the negative relationship became statistically non-significant (Fig. 5C).

## 4 Discussion

This study investigated the longitudinal patterns of microstructural alterations in cerebellar peduncles during the progression of PD and their clinical correlates. Our main findings are that i) Microstructural integrity metrics of cerebellar peduncles changed dynamically as disease progressed, shifting from an initial increased integrity to an ultimate decline. ii) The volume loss in specific cerebellar lobular regions correlated with an augmentation in the microstructural integrity metrics of cerebellar peduncles. iii) As striatal dopaminergic degeneration occurred, cerebellar peduncles integrity metrics initially increased, but this relationship became non-significant beyond a certain point, suggesting a potential loss of compensatory mechanisms. iv) The relationship between microstructural alterations in cerebellar peduncles and clinical presentations may vary across different stages of the disease. Overall, our results suggest a non-linear evolution of microstructural integrity in cerebellar peduncles throughout the course of PD. In the early-stage, increased cerebellar white matter integrity metrics were associated with cerebellar volume loss, dopaminergic degeneration, and worsening clinical symptoms. These results may reflect an early-stage adaptive structural reorganization of the cerebellum with progressive basal ganglia dysfunction in PD.

For longitudinal analyses, in the initial 2-year follow-up period, we observed an increase in FAt and a decrease in MDt over time in cerebellar peduncles, while during the subsequent 2–4 years of follow-up, we noticed the opposite trend. Our findings align with a recent cross-sectional study in PD patients using fixel-based analysis, showing stage-specific changes in white matter integrity (Li et al., 2020). As PD progresses, white matter integrity in the SCP may increase, potentially as a compensatory response to motor deficits (Li et al., 2020). Enhanced integrity in cerebellar tracts may have facilitated improved information transmission between brain regions and integration within the cerebellum, particularly in the early stage of the disease. Consistently, post-mortem studies of cerebellar pathology in PD have revealed an increase in synaptic connections between afferent fibers and Purkinje cells (Kuo et al., 2017). PD patients with resting tremor were also found to exhibit increased white matter integrity in the cerebellum, suggesting possible compensatory mechanism in response to abnormal dopamine-dependent circuits (Xiao et al., 2021, Zhong et al., 2023). In our study, the lack of significant alterations in diffusion metrics in the cerebellar peduncles over the entire four-year period may be due to temporary changes, where a decline in FAt during years 2–4 was offset by an increase in FAt during years 0–2. These observations suggest that cerebellar white matter variations in diffusion MRI-derived indices are neither static nor monotonic. Previous research has proposed that a dynamic balance may exist within the white matter microenvironment in PD (Rau et al., 2019), potentially explaining the inconsistent findings observed in cross-sectional or short-term longitudinal studies (Taylor et al., 2018, Li et al., 2020, Atkinson-Clement et al., 2017).

Compensatory mechanisms in PD could be also interpreted beyond the cerebellum. Non-linear longitudinal changes in specific white matter integrity have already been identified, displaying variations over time and in different regions, such as the corpus callosum (Rau et al., 2019). However, due to limited brain coverage in MRI acquisition, the cerebellum was not investigated in this study (Rau et al., 2019). We further extend these findings to the cerebellum, revealing a similar temporary changing pattern. Recent cross-sectional study has also shown increased white matter integrity metrics in the corticospinal tract during the early stages of PD, crucial for motor signal transmission from the brain to the spinal cord (Xiao et al., 2021, Andica et al., 2021). Therefore, exploring the interconnections among cerebral regions and the cerebellum in future investigations could offer a more comprehensive understanding of adaptive structural reorganization in PD.

We investigated cerebellar volume loss and their alignment with changes in cerebellar peduncles. Different stages exhibited distinct cerebellar volume reduction patterns, with early atrophy notably present in Crus II. Both baseline and initial two-year analyses revealed a correlation between cerebellar volume loss in Crus II and increased microstructural integrity in cerebellar peduncles. The coexistence of brain volume decline and compensatory mechanisms has been revealed in prior models of neurodegenerative diseases during the earliest phase (Gregory et al., 2018). However, during the 2 to 4-year follow-up period, we did not observe this correspondence. Recent findings highlight significant anatomical connections between the cerebellum and basal ganglia (Bostan and Strick, 2010), with Crus II identified as a key area in the cerebellum receiving projections from the basal ganglia. Additionally, it has been proposed that the activation of the cerebellum may represent a compensatory mechanism in response to striatum-cortical dysfunction (Hallett and Wu, 2013).

Then, we explored the relationship between cerebellar white matter integrity and dopaminergic degeneration in the striatum and found that in the early stage, as dopaminergic degeneration progressed, cerebellar white matter FAt increased. However, this relationship was not observed at 4-year follow-up, suggesting that the relationship between white matter integrity and dopaminergic degeneration is nonlinear and varies at different stages. Furthermore, our two-lines analysis revealed the potential existence of a threshold for dopaminergic degeneration. When degeneration did not reach this threshold, white matter FAt increased, possibly as a compensatory mechanism. However, when degeneration reached a certain degree, this compensatory effect might fail to operate. Therefore, early increased white matter integrity in the cerebellar peduncles could be considered as an initial and effective compensatory response to the underlying pathology. Previous research has consistently shown that persistent activity of Purkinje cells in the cerebellum is related to dopaminergic neuronal loss in PD, consistently supporting the potential compensatory role of the cerebellum in PD pathology (Heman et al., 2012). Indeed, an underlying compensation model has been proposed to explain the dynamic changes in neurodegenerative diseases (Gregory et al., 2017). This model posits that increasing brain activation sustains performance in response to neuronal and functional decline, but with disease progression, a subsequent phase of decompensation occurs. A recent animal study suggests that the cerebellum may play a compensatory role in PD through direct connections with the basal ganglia, specifically via the cerebellum-to-substantia nigra pars compacta pathway, which could help maintain dopamine levels (Washburn et al., 2024). Our study hypothesizes that the increased microstructural integrity of cerebellar projections may reflect a compensatory response to nigrostriatal degeneration, supported by clinical evidence.

In addition, we also observed a relationship at both baseline and 2-year follow-up in PD, but not in the 4-year, where the worsening of RBD symptoms was associated with an increase in cerebellar white matter integrity. This aligns with earlier studies highlighting increased cerebellar metabolism and functional activation, and their associations with the severity of RBD symptoms in PD (Meles et al., 2021, Liu et al., 2021). However, it is important to note that this result does not provide direct evidence, as we did not find a correlation between primary motor symptoms (e.g., UPDRS Part III) and cerebellar peduncle white matter integrity.

Animal studies have shown that stimulations in cerebellum can enhance the spontaneous compensatory capacities of the cerebellum to counter deficits caused by brain injury or neurodegeneration (Gelfo and Petrosini, 2022). Additionally, it has been discovered that the cerebellum could undergo plastic rearrangements to compensate for deficits caused by lesions (Sadeghihassanabadi et al., 2022, Gelfo and Petrosini, 2022). Of note, regardless of the supportive evidence, caution is still warranted when interpreting our results. For example, increased integrity metrics in cerebellar peduncles in early-stage PD is expected to be associated with the mitigation of neurodegeneration or slower clinical deterioration, thus directly supporting the indicative of “cerebellar reserve”, a recently proposed concept, emphasizing the highly compensatory nature of the cerebellum (Mitoma et al., 2020). In contrast, in our study the early-stage increased integrity in cerebellar peduncles, possibly attributable to volume loss, is associated with dopaminergic degeneration and clinical deterioration. Although our findings align with previous works on compensatory models of degenerative diseases, supported by increased compensatory activity in individuals with a higher disease burden (Gregory et al., 2018, Rittman et al., 2019), we should acknowledge the fact that the underlying mechanisms involved in compensation in PwPD are multifactorial and could be attributed to different pathophysiology, clinical staging and protective factors (Gregory et al., 2018, Stern et al., 2020). Still, our findings pave the way for future investigations to unveil the underlying mechanism and assess whether individuals with stronger compensatory function could exhibit better long-term clinical outcomes. In addition, it can help to identify effective interventions to enhance compensatory effects (Yoo et al., 2019, Billeri and Naro, 2021).

Our study has two key strengths. Firstly, we employed state-of-the-art methods for accurately reconstructing cerebellar peduncles while effectively correcting for CSF partial volume, ensuring the reliability of our findings. Secondly, we observed non-linear changes in the microstructural integrity of the cerebellar peduncles over a four-year follow-up, with trends in the initial two-year period supported by two subset analyses. While the concept of compensation should be interpreted cautiously, we believe this finding lays important groundwork for future studies exploring the compensatory mechanisms of the cerebellum in PD.

This study has some limitations. First, methodological constraints related to diffusion scanning should be acknowledged. The relatively low resolution and limited number of diffusion directions may result in imprecise estimation of diffusivity measures. Although our model effectively accounts for the influence of CSF on DWI signals, it does not fully capture the diffusion characteristics of extracellular water in the spaces surrounding neurons and glial cells. Second, we did not specifically address the issue of laterality, despite its importance, as PD can lead to both tissue degeneration and potential reorganization in the early stages, which may differ depending on the laterality of motor symptoms (Xiao et al., 2021). The laterality of cerebellar peduncle dominance and its associated imaging features is a complex issue that merits further exploration. Third, our study did not include the dentate nucleus in the cerebellar gray matter volumetric analysis, despite its critical role in cerebellar and basal ganglia interactions. Further studies focusing on the dentate nucleus and its connections with other strategic regions will provide deeper insights into the role of cerebellum in PD.

In conclusion, our study suggests non-linear dynamic changes in the microstructural integrity of cerebellar peduncles in PD over time, with varying relationships with clinical symptoms across disease stages. Early increased white matter integrity metrics was associated with cerebellar volume loss, dopaminergic degeneration, and worsening clinical symptoms. These findings support the compensatory role of cerebellar peduncles in response to PD pathology in early stage. Our findings provide insights into cerebro-cerebellar communications at different stages, highlighting the importance of considering the interplay between the cerebellum and disease progression. A deeper understanding of the mechanisms underlying cerebellar compensatory role may be crucial in developing therapeutic interventions for PD patients.

## Funding

This work was supported by 10.13039/501100001809National Natural Science Foundation of China (No. 82071419, No. 82301663, No. 82301420); Key Research and Development Program of Guangzhou (No. 202206010086); Science and Technology Planning Project of Guangzhou (No. 202201000005); 10.13039/501100002858China Postdoctoral Science Foundation (No. 2023M730742); Guangdong Provincial Key Laboratory of Artificial Intelligence in Medical Image Analysis and Application (No. 2022B1212010011).

## CRediT authorship contribution statement

**Chentao He:** Writing – review & editing, Writing – original draft, Visualization, Software, Methodology, Investigation, Funding acquisition, Formal analysis, Data curation. **Rui Yang:** Writing – review & editing, Writing – original draft, Methodology, Investigation, Formal analysis, Conceptualization. **Siming Rong:** Writing – review & editing, Writing – original draft, Methodology. **Piao Zhang:** Data curation, Conceptualization. **Xi Chen:** Writing – review & editing, Writing – original draft, Investigation, Formal analysis, Conceptualization. **Qi Qi:** Data curation. **Ziqi Gao:** Formal analysis, Data curation. **Yan Li:** Formal analysis, Data curation. **Hao Li:** Writing – review & editing. **Frank-Erik de Leeuw:** Writing – review & editing. **Anil M. Tuladhar:** Writing – review & editing. **Marco Duering:** Writing – review & editing. **Rick C. Helmich:** Writing – review & editing, Conceptualization. **Rick van der Vliet:** Writing – review & editing. **Sirwan K.L. Darweesh:** Writing – review & editing, Supervision, Conceptualization. **Zaiyi Liu:** Supervision, Project administration, Funding acquisition. **Lijuan Wang:** Writing – review & editing, Supervision, Funding acquisition, Data curation, Conceptualization. **Mengfei Cai:** Writing – review & editing, Writing – original draft, Methodology, Funding acquisition, Formal analysis, Data curation, Conceptualization. **Yuhu Zhang:** Writing – review & editing, Writing – original draft, Visualization, Validation, Supervision, Software, Resources, Project administration, Methodology, Investigation, Funding acquisition, Data curation, Conceptualization.

## Declaration of competing interest

The authors declare that they have no known competing financial interests or personal relationships that could have appeared to influence the work reported in this paper.

## Data Availability

Data will be made available on request.
