# Supplementary material for: Temporal evolution of microstructural integrity in cerebellar peduncles in Parkinson’s disease: Stage-specific patterns and dopaminergic correlates
Source: Neuroimage Clin. 2024 Sep 29;44:103679. doi: 10.1016/j.nicl.2024.103679 (PMC11489329; doi:10.1016/j.nicl.2024.103679)
Supplement: Supplementary Data 1 [file mmc1.docx]

**Supplementary materials**


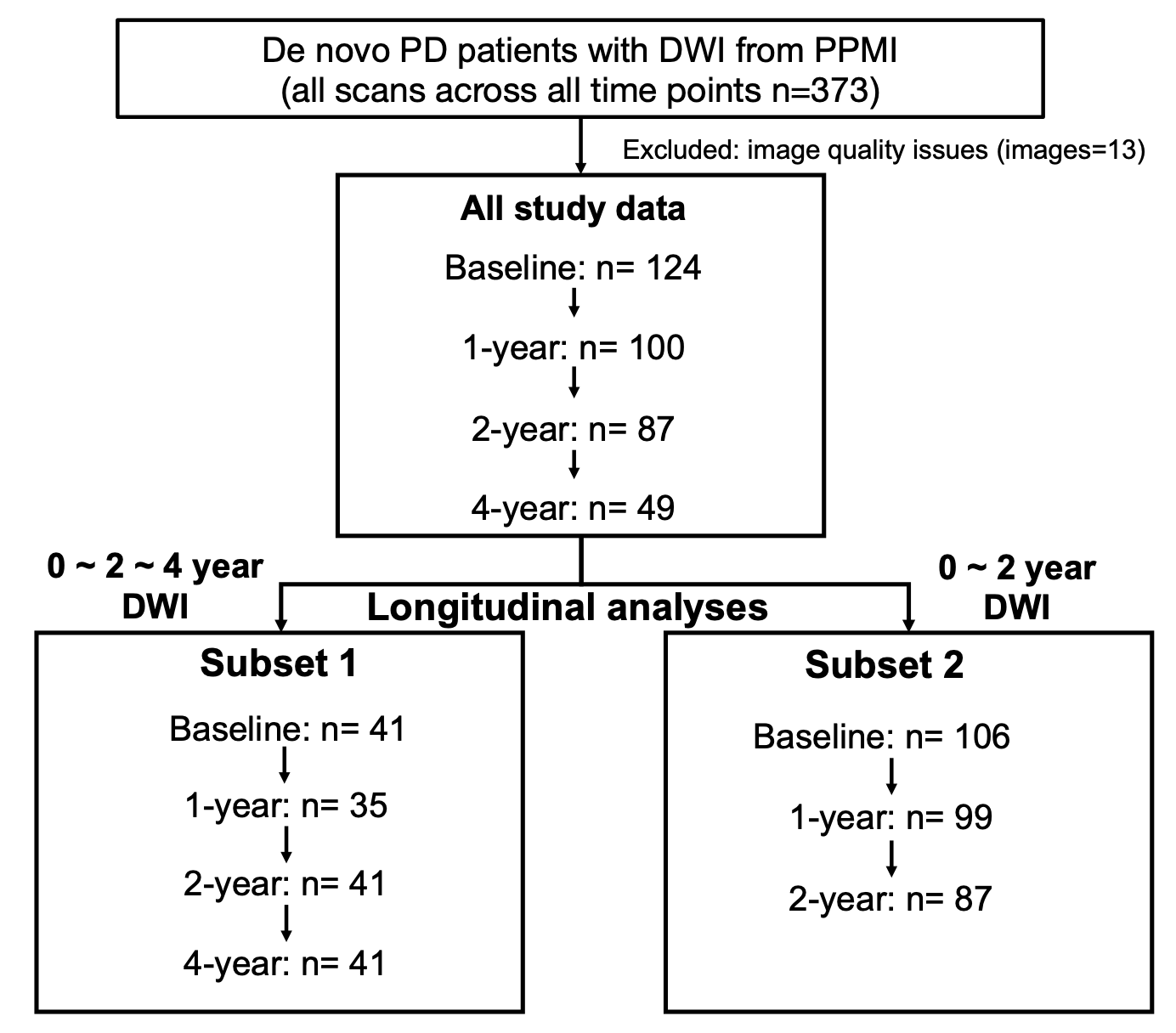


**Supplementary Figure 1:** Flowchart of the study population


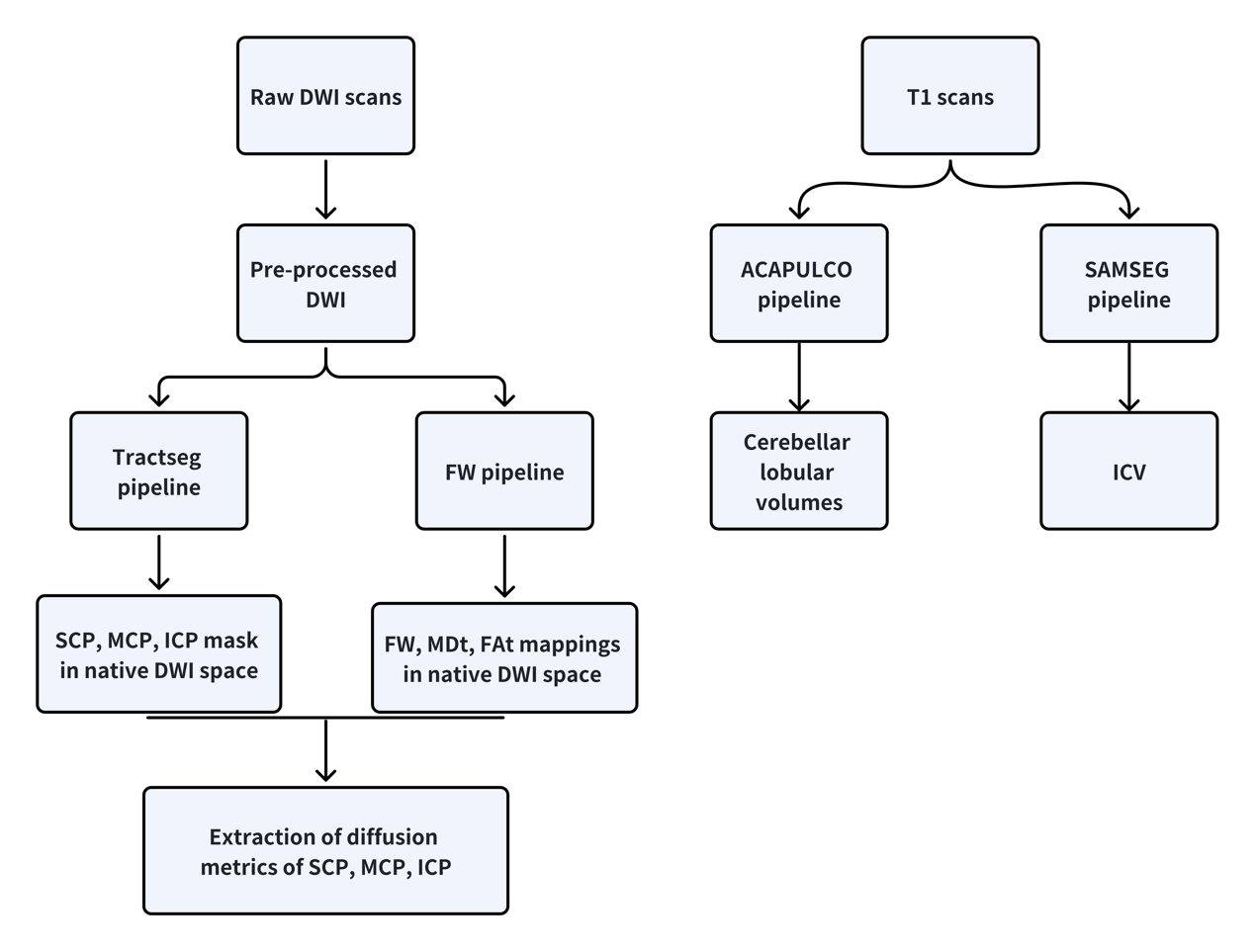


**Supplementary Figure 2:** Workflow of MRI processing. Raw DWI scans were pre-processed, followed by the Tractseg pipeline to obtain the SCP, MCP and ICP masks and the free water pipeline to derive the diffusion mappings. Then the diffusion metrics (FW, MDt, FAt) were extracted in the native space. T1 scans were processed by ACAPULCO pipeline to obtain cerebellar lobular volumes and by the SAMSEG tool to obtain the intracranial volume. Of note, this workflow was used for the MRI scans at each time point, since these scans of each participant were acquired from the identical scanners and MRI acquisition protocols, no within-subject co-registration was used. ICP = Inferior Cerebellar Peduncle; MCP = Middle Cerebellar Peduncle; SCP = Superior Cerebellar Peduncle;

MDt = free water-corrected mean diffusivity; FAt = free water-corrected fractional anisotropy; FW = free water; TractSeg = Tractography-based Segmentation; ACAPULCO = Automatic Cerebellum Anatomical Parcellation using U-Net with Locally Constrained Optimization; SAMSEG = Sequence Adaptive Multimodal SEGmentation; ICV = intracranial volume.


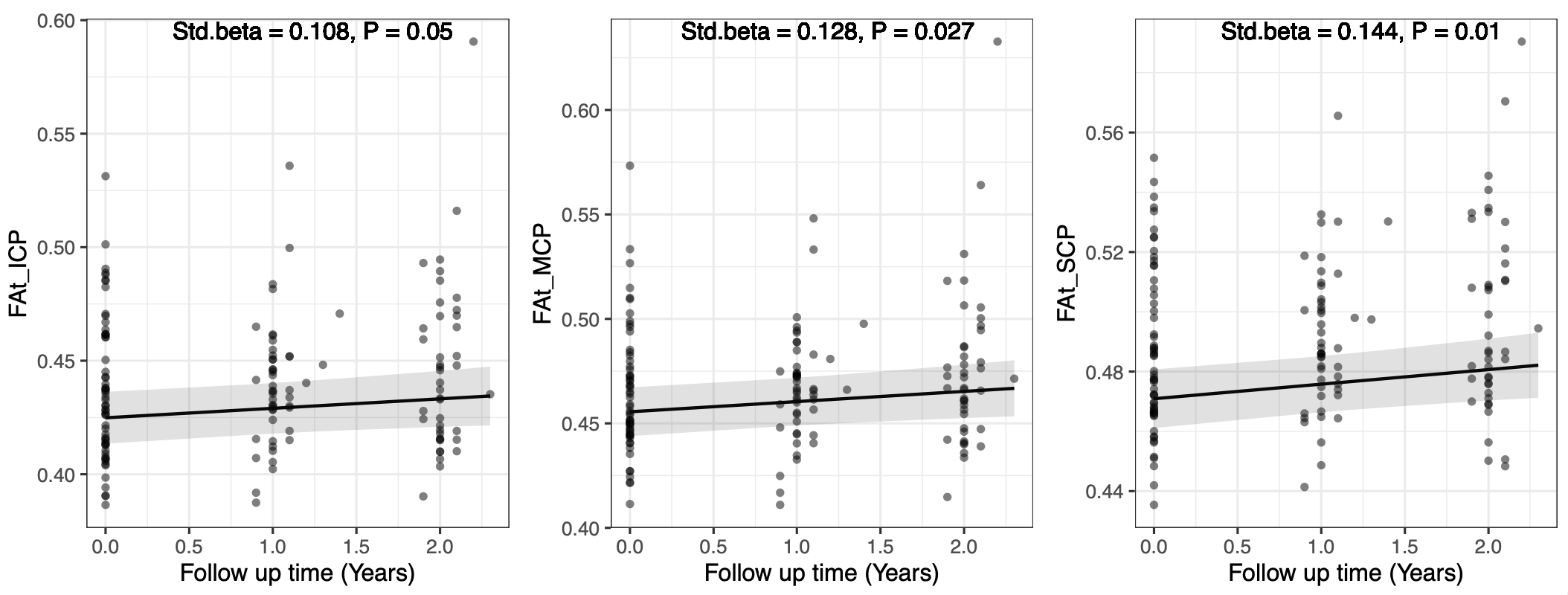


**Supplementary Figure 3:** Longitudinal changes of microstructural integrity in cerebellar peduncles in patients who had baseline scan within 1.5 year of disease onset (n=60) to confirm the FAt increase in the following two years. ICP = Inferior Cerebellar Peduncle; MCP = Middle Cerebellar Peduncle; SCP = Superior Cerebellar Peduncle; FAt = free water-corrected fractional anisotropy; Std.beta = Standardized beta coefficients.

**
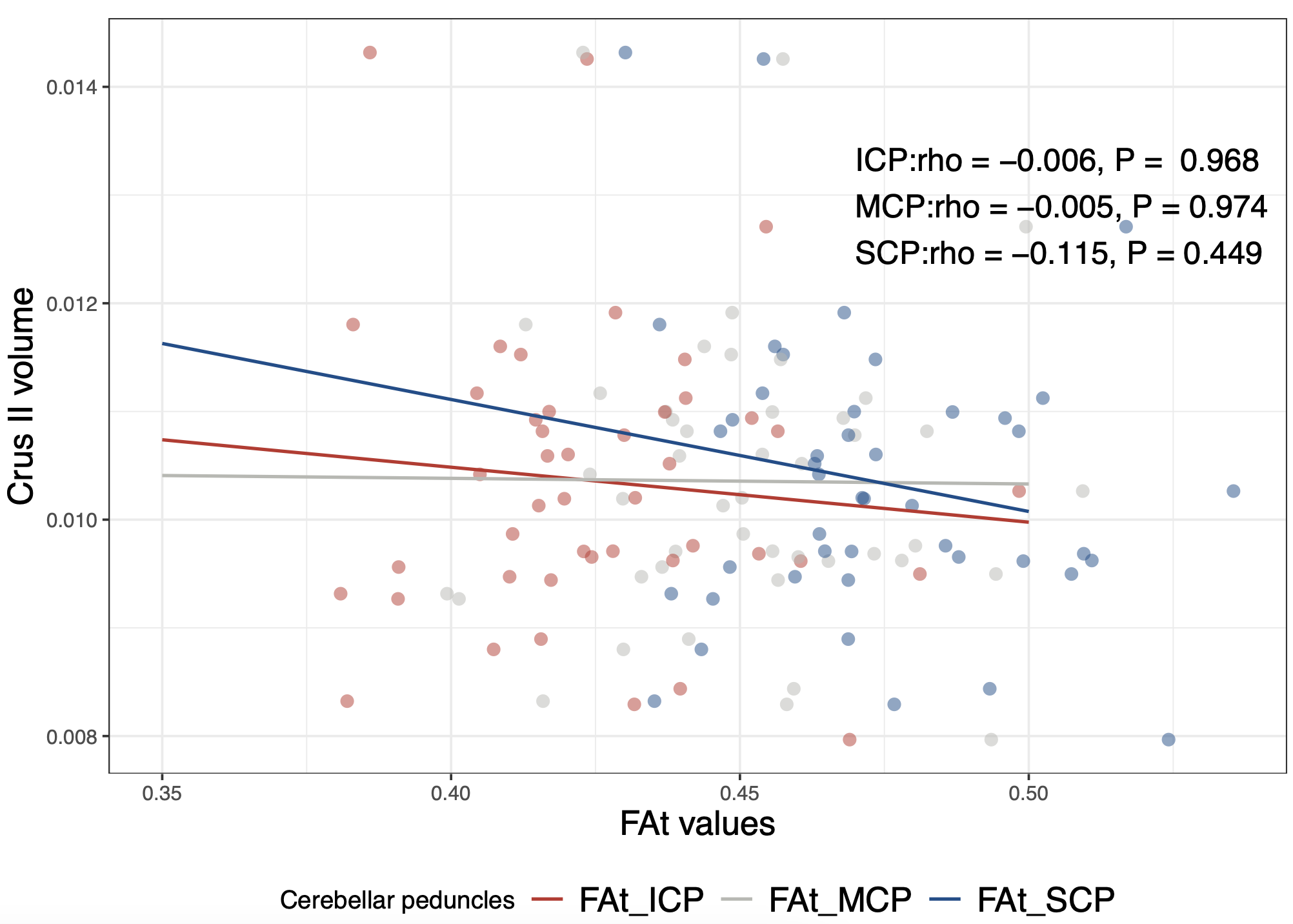
**

**Supplementary Figure 4:** Correlation between cerebellar volumes and microstructural metrics in cerebellar peduncles in the control group. At baseline (n=45), no significant correlation was observed between the microstructural integrity of the cerebellar peduncles and the volumes of cerebellar lobule Crus II in the control group. ICP = Inferior Cerebellar Peduncle; MCP = Middle Cerebellar Peduncle; SCP = Superior Cerebellar Peduncle; FAt = free water-corrected fractional anisotropy.


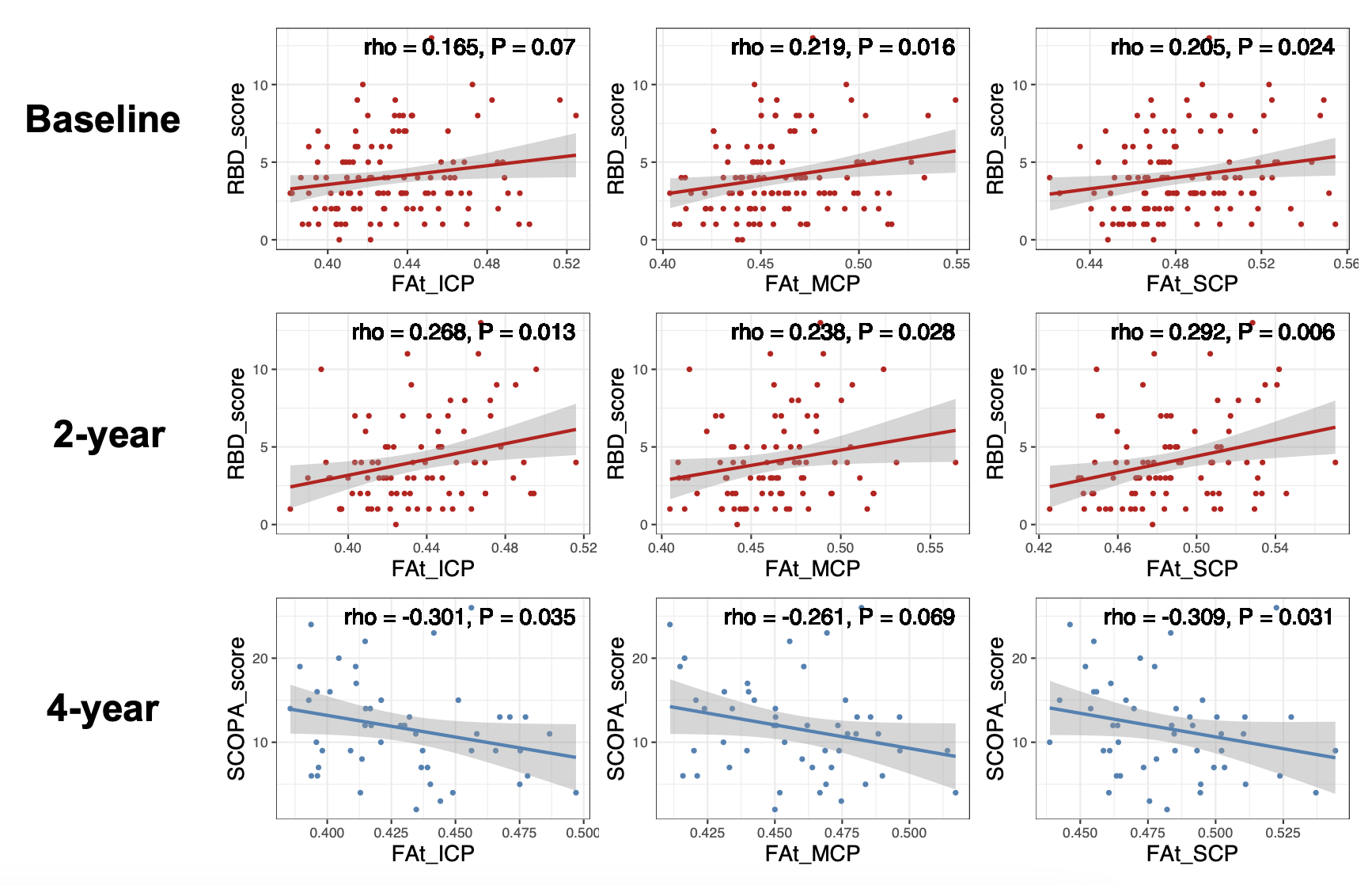


**Supplementary Figure 5:** Correlations between cerebellar diffusion metrics and clinical symptoms in PD at baseline, 2-year, and 4-year follow-up. ICP = Inferior Cerebellar Peduncle; MCP = Middle Cerebellar Peduncle; SCP = Superior Cerebellar Peduncle; MDt = free water-corrected mean diffusivity; RBD = REM Sleep Behavior Questionnaire; SCOPA-AUT = Scales for Outcomes in Parkinson’s disease - Autonomic Dysfunction.

**Supplementary Table 1:** Site information and participant distribution across study subsets.

| **Subset 1** | | **Subset 2** | |
| --- | --- | --- | --- |
| Site number | Number of Participants | Site number | Number of Participants |
| 13 | 10 | 13 | 16 |
| 14 | 2 | 14 | 3 |
| 19 | 2 | 18 | 1 |
| 23 | 5 | 19 | 2 |
| 25 | 8 | 23 | 13 |
| 52 | 14 | 25 | 17 |
|  |  | 28 | 17 |
|  |  | 33 | 1 |
|  |  | 51 | 17 |
|  |  | 52 | 19 |

**Supplementary Table 2.** Longitudinal changes of microstructural integrity in cerebellar peduncles over time.

|  | **Subset 1 (N=41)** | | | | **Subset 2 (N=106)** | |
| --- | --- | --- | --- | --- | --- | --- |
|  | **baseline to year 2** | | **year 2 to year 4** | | **baseline to year 2** | |
| Variables | Estimate | P | Estimate | P | Estimate | P |
| FAt_ICP | 0.107 | 0.064 | -0.178 | 0.004* ^a^ | 0.091 | 0.018* |
| FAt_MCP | 0.141 | 0.011* | -0.216 | 0.001* ^a^ | 0.096 | 0.012* |
| FAt_SCP | 0.136 | 0.011* | -0.167 | 0.016* | 0.113 | 0.002* ^a^ |
| MDt_ICP | -0.101 | 0.054 | 0.208 | 0.004* ^a^ | -0.112 | 0.005* ^a^ |
| MDt_MCP | -0.122 | 0.024* | 0.233 | 0.001* ^a^ | -0.101 | 0.013* |
| MDt_SCP | -0.126 | 0.018* | 0.182 | 0.017* | -0.11 | 0.008* |
| FWf_ICP | 0.006 | 0.915 | -0.014 | 0.868 | 0.018 | 0.655 |
| FWf_MCP | 0.003 | 0.956 | -0.027 | 0.689 | -0.002 | 0.964 |
| FWf_SCP | -0.028 | 0.591 | 0.008 | 0.883 | -0.028 | 0.422 |

ICP = Inferior Cerebellar Peduncle; MCP = Middle Cerebellar Peduncle; SCP = Superior Cerebellar Peduncle; MDt = free water-corrected mean diffusivity; FAt = free water-corrected fractional anisotropy; FWf = free water fraction, Estimate = Standardized beta coefficients. Estimate denotes the progression rate of each metrics extracted from linear-mixed effects model. * represents uncorrected P-values < 0.05. ^a^ indicates statistical significance remained after Bonferroni multiple comparison correction.

**Supplementary Table 3.** Longitudinal changes in the microstructural integrity of cerebellar peduncles from baseline to 2-year follow-up, adjusted for total levodopa equivalent daily dose (LEDD).

|  | **Subset 1 (N=41)** | | **Subset 2 (N=106)** | |
| --- | --- | --- | --- | --- |
| Variables | Estimate | P | Estimate | P |
| FAt_ICP | 0.106 | 0.066 | 0.125 | 0.001* |
| FAt_MCP | 0.141 | 0.011* | 0.132 | 0.001* |
| FAt_SCP | 0.136 | 0.011* | 0.064 | 0.259 |
| MDt_ICP | -0.101 | 0.054 | -0.096 | 0.019* |
| MDt_MCP | -0.122 | 0.024* | -0.102 | 0.01* |
| MDt_SCP | -0.126 | 0.018* | -0.118 | 0.062 |
| FWf_ICP | 0.006 | 0.914 | -0.038 | 0.427 |
| FWf_MCP | 0.003 | 0.956 | -0.055 | 0.201 |
| FWf_SCP | -0.028 | 0.59 | -0.07 | 0.157 |

ICP = Inferior Cerebellar Peduncle; MCP = Middle Cerebellar Peduncle; SCP = Superior Cerebellar Peduncle; MDt = free water-corrected mean diffusivity; FAt = free water-corrected fractional anisotropy; FWf = free water fraction, Estimate = Standardized beta coefficients. Estimate denotes the progression rate of each metrics extracted from linear-mixed effects model.

**Supplementary Table 4.** Microstructural changes in cerebellar peduncles from baseline to 4 years in subset 1 (N=41).

| Variables | Estimate | P |
| --- | --- | --- |
| FAt_ICP | -0.048 | 0.347 |
| FAt_MCP | -0.044 | 0.39 |
| FAt_SCP | -0.013 | 0.798 |
| MDt_ICP | 0.076 | 0.202 |
| MDt_MCP | 0.074 | 0.199 |
| MDt_SCP | 0.035 | 0.544 |
| FWf_ICP | -0.017 | 0.748 |
| FWf_MCP | -0.041 | 0.379 |
| FWf_SCP | -0.034 | 0.408 |

ICP = Inferior Cerebellar Peduncle; MCP = Middle Cerebellar Peduncle; SCP = Superior Cerebellar Peduncle; MDt = free water-corrected mean diffusivity; FAt = free water-corrected fractional anisotropy; FWf = free water fraction. Estimate denotes the progression rate of each metrics extracted from linear-mixed effects model.

**Supplementary Table 5.** Correlation coefficients between diffusion metrics in cerebellar peduncles and volumes of cerebellar lobules at baseline (n=124) at baseline.

| volumes of cerebellar lobules | MDt_ICP | MDt_MCP | MDt_SCP | FAt_ICP | FAt_MCP | FAt_SCP |
| --- | --- | --- | --- | --- | --- | --- |
| I-III | 0.192* | 0.207* | 0.192* | -0.164 | -0.177 | -0.139 |
| IV | 0.074 | 0.075 | 0.096 | -0.074 | -0.019 | -0.116 |
| V | -0.037 | -0.021 | -0.038 | -0.058 | -0.043 | -0.084 |
| VI | 0.038 | 0.078 | 0.038 | -0.099 | -0.157 | -0.031 |
| Crus I | -0.012 | 0.026 | 0.022 | 0.019 | -0.016 | -0.052 |
| Crus II | 0.172 | 0.188* | 0.194* | -0.276* | -0.275* | -0.288* |
| VIIb | 0.102 | 0.122 | 0.142 | -0.181* | -0.139 | -0.188* |
| VIIIa | 0.068 | 0.045 | 0.069 | -0.051 | -0.05 | 0.032 |
| VIIIb | 0.111 | 0.103 | 0.113 | -0.066 | -0.055 | -0.066 |
| IX | 0.166 | 0.162 | 0.157 | -0.144 | -0.102 | -0.122 |
| X | 0.051 | 0.037 | 0.05 | 0.029 | 0.019 | 0.062 |
| Vermis | 0.116 | 0.157 | 0.161 | -0.203* | -0.209* | -0.218* |

ICP = Inferior Cerebellar Peduncle; MCP = Middle Cerebellar Peduncle; SCP = Superior Cerebellar Peduncle; MDt = free water-corrected mean diffusivity; FAt = free water-corrected fractional anisotropy. * indicates significant correlations.

**Supplementary Table 6.** Correlation coefficients between slopes of diffusion metrics in cerebellar peduncles and cerebellar lobules atrophy rate from baseline to 2 follow-up years in subset 1 (n=41).

| cerebellar lobules atrophy rate | MDt_ICP slope | MDt_MCP slope | MDt_SCP slope | FAt_ICP slope | FAt_MCP slope | FAt_SCP slope |
| --- | --- | --- | --- | --- | --- | --- |
| I-III | 0.061 | 0.078 | 0.027 | -0.005 | 0.006 | 0.073 |
| IV | -0.181 | -0.17 | -0.153 | 0.118 | 0.125 | 0.117 |
| V | 0.063 | 0.09 | 0.098 | -0.111 | -0.197 | -0.096 |
| VI | 0.176 | 0.102 | 0.097 | -0.026 | 0.01 | 0.134 |
| Crus I | -0.098 | -0.053 | -0.099 | 0.085 | 0.021 | 0.017 |
| Crus II | 0.359* | 0.333* | 0.402* | -0.418* | -0.345* | -0.448* |
| VIIb | 0.004 | 0.054 | -0.022 | -0.076 | -0.192 | -0.082 |
| VIIIa | -0.126 | -0.126 | -0.08 | 0.117 | 0.068 | 0.04 |
| VIIIb | -0.035 | -0.021 | -0.029 | -0.165 | -0.177 | -0.098 |
| IX | -0.141 | -0.169 | -0.156 | 0.088 | -0.036 | 0.016 |
| X | 0.077 | 0.038 | 0.054 | 0.082 | 0.065 | 0.14 |
| Vermis | -0.073 | -0.057 | -0.075 | 0.021 | -0.054 | 0.019 |

ICP = Inferior Cerebellar Peduncle; MCP = Middle Cerebellar Peduncle; SCP = Superior Cerebellar Peduncle; MDt = free water-corrected mean diffusivity; FAt = free water-corrected fractional anisotropy. * indicates significant correlations.

**Supplementary Table 7.** Correlation coefficients between slopes of diffusion metrics in cerebellar peduncles and cerebellar lobules atrophy rate from 2 to 4 follow-up years in subset 1(n=41).

| cerebellar lobules atrophy rate | MDt_ICP slope | MDt_MCP slope | MDt_SCP slope | FAt_ICP slope | FAt_MCP slope | FAt_SCP slope |
| --- | --- | --- | --- | --- | --- | --- |
| I-III | -0.199 | -0.25 | -0.295 | 0.104 | 0.038 | 0.138 |
| IV | 0.048 | 0.081 | 0.208 | 0.034 | -0.013 | -0.202 |
| V | -0.16 | -0.157 | -0.142 | 0.077 | 0.012 | 0.102 |
| VI | -0.03 | 0.039 | 0.025 | 0.016 | -0.174 | -0.06 |
| Crus I | 0.079 | -0.005 | 0.066 | -0.063 | -0.196 | -0.089 |
| Crus II | 0.198 | 0.215 | 0.249 | -0.071 | -0.076 | -0.227 |
| VIIb | 0.057 | 0.161 | 0.208 | -0.133 | -0.19 | -0.27 |
| VIIIa | 0.193 | 0.248 | 0.209 | -0.223 | -0.126 | -0.194 |
| VIIIb | 0.003 | -0.027 | 0.116 | 0.113 | 0.213 | 0.095 |
| IX | 0.01 | 0.111 | 0.074 | 0.18 | 0.057 | 0.047 |
| X | 0.169 | 0.215 | 0.161 | -0.125 | -0.328* | -0.223 |
| Vermis | -0.216 | -0.155 | -0.06 | 0.199 | 0.088 | -0.021 |

ICP = Inferior Cerebellar Peduncle; MCP = Middle Cerebellar Peduncle; SCP = Superior Cerebellar Peduncle; MDt = free water-corrected mean diffusivity; FAt = free water-corrected fractional anisotropy. *Significant Correlations Indicated.

**Supplementary Table 8.** Clinical correlations with microstructural integrity in cerebellar peduncles at different follow-up time points (baseline, 2-year, and 4-year) in PD.

|  |  | Baseline | | 2-year | | 4-year | |
| --- | --- | --- | --- | --- | --- | --- | --- |
| Metrics_peduncles | Clinical score | rho | P | rho | P | rho | P |
| FAt_ICP | MDS-UPDRS-I | 0 | 0.998 | 0.02 | 0.88 | -0.25 | 0.087 |
| FAt_MCP | MDS-UPDRS-I | 0.04 | 0.699 | 0.05 | 0.665 | -0.22 | 0.126 |
| FAt_SCP | MDS-UPDRS-I | 0.05 | 0.571 | 0.08 | 0.472 | -0.15 | 0.311 |
| FAt_ICP | MDS-UPDRS-II | 0.11 | 0.22 | 0.15 | 0.176 | -0.01 | 0.924 |
| FAt_MCP | MDS-UPDRS-II | 0.06 | 0.524 | 0.08 | 0.448 | -0.05 | 0.744 |
| FAt_SCP | MDS-UPDRS-II | 0.16 | 0.082 | 0.22 | 0.041* | 0.07 | 0.653 |
| FAt_ICP | MDS-UPDRS-III | 0.02 | 0.826 | 0.13 | 0.363 | 0.05 | 0.743 |
| FAt_MCP | MDS-UPDRS-III | -0.01 | 0.888 | 0.09 | 0.538 | 0 | 0.998 |
| FAt_SCP | MDS-UPDRS-III | 0.06 | 0.546 | 0.11 | 0.452 | 0.05 | 0.733 |
| FAt_ICP | GDS-15 | -0.01 | 0.941 | -0.1 | 0.377 | -0.03 | 0.828 |
| FAt_MCP | GDS-15 | 0.03 | 0.766 | -0.09 | 0.402 | -0.06 | 0.675 |
| FAt_SCP | GDS-15 | 0.04 | 0.68 | 0.02 | 0.886 | 0.02 | 0.883 |
| FAt_ICP | STAI | 0.01 | 0.882 | 0.14 | 0.216 | -0.06 | 0.677 |
| FAt_MCP | STAI | 0.07 | 0.433 | 0.14 | 0.216 | -0.15 | 0.3 |
| FAt_SCP | STAI | 0.1 | 0.268 | 0.16 | 0.154 | -0.1 | 0.49 |
| FAt_ICP | MoCA | 0.1 | 0.285 | -0.11 | 0.296 | 0.06 | 0.689 |
| FAt_MCP | MoCA | 0.05 | 0.601 | -0.13 | 0.224 | 0.06 | 0.673 |
| FAt_SCP | MoCA | 0.04 | 0.692 | -0.26 | 0.017* | -0.03 | 0.856 |
| FAt_ICP | SCOPA-AUT | 0.13 | 0.152 | 0.12 | 0.256 | -0.3 | 0.035* |
| FAt_MCP | SCOPA-AUT | 0.13 | 0.155 | 0.11 | 0.297 | -0.26 | 0.069 |
| FAt_SCP | SCOPA-AUT | 0.1 | 0.267 | 0.15 | 0.183 | -0.31 | 0.031* |
| FAt_ICP | RBD | 0.17 | 0.071 | 0.27 | 0.013* | 0.19 | 0.195 |
| FAt_MCP | RBD | 0.22 | 0.016* | 0.24 | 0.028* | 0.19 | 0.183 |
| FAt_SCP | RBD | 0.21 | 0.025* | 0.29 | 0.007* | 0.18 | 0.216 |
| MDt_ICP | MDS-UPDRS-I | 0.03 | 0.71 | -0.02 | 0.833 | 0.28 | 0.051 |
| MDt_MCP | MDS-UPDRS-I | -0.02 | 0.856 | -0.05 | 0.659 | 0.27 | 0.06 |
| MDt_SCP | MDS-UPDRS-I | 0.02 | 0.847 | -0.08 | 0.441 | 0.32 | 0.026* |
| MDt_ICP | MDS-UPDRS-II | 0.07 | 0.467 | -0.09 | 0.407 | 0.03 | 0.819 |
| MDt_MCP | MDS-UPDRS-II | 0.06 | 0.496 | -0.02 | 0.877 | 0 | 0.98 |
| MDt_SCP | MDS-UPDRS-II | 0.07 | 0.452 | -0.04 | 0.737 | 0 | 0.988 |
| MDt_ICP | MDS-UPDRS-III | 0.11 | 0.245 | -0.09 | 0.502 | -0.05 | 0.73 |
| MDt_MCP | MDS-UPDRS-III | 0.11 | 0.225 | -0.01 | 0.97 | -0.08 | 0.602 |
| MDt_SCP | MDS-UPDRS-III | 0.14 | 0.129 | 0.05 | 0.706 | -0.08 | 0.607 |
| MDt_ICP | GDS-15 | 0.02 | 0.791 | 0.07 | 0.507 | 0.09 | 0.519 |
| MDt_MCP | GDS-15 | 0 | 0.964 | 0.06 | 0.587 | 0.07 | 0.643 |
| MDt_SCP | GDS-15 | -0.04 | 0.698 | 0.03 | 0.77 | 0.07 | 0.628 |
| MDt_ICP | STAI | 0.08 | 0.387 | -0.1 | 0.375 | 0.1 | 0.486 |
| MDt_MCP | STAI | 0 | 0.98 | -0.14 | 0.216 | 0.12 | 0.426 |
| MDt_SCP | STAI | 0.04 | 0.632 | -0.11 | 0.324 | 0.13 | 0.384 |
| MDt_ICP | MoCA | 0.01 | 0.891 | 0.1 | 0.374 | -0.17 | 0.243 |
| MDt_MCP | MoCA | 0.04 | 0.661 | 0.14 | 0.189 | -0.13 | 0.358 |
| MDt_SCP | MoCA | -0.01 | 0.928 | 0.17 | 0.116 | -0.14 | 0.352 |
| MDt_ICP | SCOPA-AUT | -0.09 | 0.319 | -0.08 | 0.493 | 0.29 | 0.043* |
| MDt_MCP | SCOPA-AUT | -0.1 | 0.295 | -0.1 | 0.345 | 0.3 | 0.037* |
| MDt_SCP | SCOPA-AUT | -0.07 | 0.446 | -0.09 | 0.438 | 0.31 | 0.03* |
| MDt_ICP | RBD | -0.09 | 0.355 | -0.22 | 0.046* | -0.12 | 0.405 |
| MDt_MCP | RBD | -0.16 | 0.09 | -0.22 | 0.042* | -0.1 | 0.482 |
| MDt_SCP | RBD | -0.12 | 0.205 | -0.21 | 0.051 | -0.09 | 0.519 |

MDS UPDRS = Movement Disorder Society - sponsored revision of the Unified Parkinson’s Disease Rating Scale; MoCA = Montreal Cognitive Assessment; GDS = Geriatric Depression Scale; STAI = State - Trait Anxiety Inventory; RBD = REM Sleep Behavior Questionnaire; SCOPA-AUT = Scales for Outcomes in Parkinson’s disease - Autonomic Dysfunction; DATSCAN = the dopamine transporter scan; ICP = Inferior Cerebellar Peduncle; MCP = Middle Cerebellar Peduncle; SCP = Superior Cerebellar Peduncle; MDt = free water-corrected mean diffusivity; FAt = free water-corrected fractional anisotropy. *Significant Correlations Indicated.

**Supplementary Table 9.** Striatal dopamine transporter specific binding ratio (DAT SBR) correlations with microstructural integrity in cerebellar peduncles at different follow-up time points (baseline, 2-year, and 4-year).

|  |  | Baseline | | 2-year | | 4-year | |
| --- | --- | --- | --- | --- | --- | --- | --- |
| Metrics_peduncles | DAT-SCAN | rho | P | rho | P | rho | P |
| FAt_ICP | Striatal_SBR | -0.21 | 0.02* | -0.2 | 0.069 | -0.25 | 0.092 |
| FAt_MCP | Striatal_SBR | -0.22 | 0.016* | -0.22 | 0.042* | -0.22 | 0.135 |
| FAt_SCP | Striatal_SBR | -0.21 | 0.023* | -0.19 | 0.081 | -0.26 | 0.078 |
| MDt_ICP | Striatal_SBR | 0.18 | 0.055 | 0.12 | 0.272 | 0.05 | 0.717 |
| MDt_MCP | Striatal_SBR | 0.22 | 0.016* | 0.21 | 0.057 | 0.1 | 0.526 |
| MDt_SCP | Striatal_SBR | 0.2 | 0.027* | 0.18 | 0.092 | 0.07 | 0.635 |

ICP = Inferior Cerebellar Peduncle; MCP = Middle Cerebellar Peduncle; SCP = Superior Cerebellar Peduncle; MDt = free water-corrected mean diffusivity; FAt = free water-corrected fractional anisotropy. *Significant Correlations Indicated.
